# Second-harmonic patterned polarization-analyzed reflection confocal microscopy of stromal collagen in benign and malignant breast tissues

**DOI:** 10.1038/s41598-018-34693-0

**Published:** 2018-11-02

**Authors:** Chukwuemeka Okoro, Varun Kelkar, Mayandi Sivaguru, Rajyasree Emmadi, Kimani C. Toussaint

**Affiliations:** 1 University of Illinois at Urbana–Champaign, PROBE Lab, Department of Electrical and Computer Engineering, Urbana, Illinois 61801 USA; 2 University of Illinois at Urbana–Champaign, Carl R. Woese Institute for Genomic Biology, Urbana, Illinois 61801 USA; 3 University of Illinois at Chicago, Department of Pathology, College of Medicine, Chicago, Illinois 60612 USA; 4 University of Illinois at Urbana–Champaign, PROBE Lab, Departments of Mechanical Science and Engineering and Bioengineering, Urbana, Illinois 61801 USA; 5 University of Illinois at Urbana–Champaign, Affiliate in the Departments of Electrical and Computer Engineering, and the Beckman Institute for Advanced Science and Technology, Urbana, Illinois 61801 USA

## Abstract

We present the results of polarimetric analysis of collagen on varying pathologies of breast tissues using second-harmonic patterned polarization-analyzed reflection confocal (SPPARC) microscopy. Experiments are conducted on a breast tissue microarray having *benign tissues* (BT), malignant *invasive lobular carcinoma* (ILC), and benign stroma adjacent to the malignant tissues (called the *benign adjacent tissue*, or BAT). Stroma in BAT and ILC exhibit the largest parameter differences. We observe that stromal collagen readings in ILC show lower depolarization, lower diattenuation and higher linear degree-of-polarization values than stromal collagen in BAT. This suggests that the optical properties of collagen change most in the vicinity of tumors. A similar trend is also exhibited in the non-collagenous extrafibrillar matrix *plus* cells (EFMC) region. The three highlighted parameters show greatest sensitivity to changes in the polarization response of collagen between pathologies.

## Introduction

Breast cancer is the most predominant cancer in women and accounts for 30% of all new female cancer cases^1^. Added to the fact that it is the second leading cause of estimated cancer-related female deaths, the importance of early diagnosis and prognosis, which leads to better survivability^2^, can be appreciated. The gold standard for cancer diagnosis on breast tissues is the qualitative assessment of hematoxylin and eosin (H&E) stained tissues performed by trained pathologists. Predicting future outcome for patients has relied on conventional prognostic factors such as tumor size, nuclear grade and axillary lymph node status^3^. Studies show that there are other prognostic markers, such as gene-expression signatures^4^ and light scattering directionality^5^, that can more accurately predict or at the very least, complement current existing criteria^6^.

Prognostic markers increasingly being recognized as important for describing growth and migration of tumors include the family of extracellular matrix (ECM) features^7–9^. In particular, the role of collagen in regulating tumor progression has been previously highlighted. For example, Keely and colleagues assigned distinctive signatures of collagen arrangement proximal to tumors as a means of classification^10^. In subsequent studies, they showed that such signatures predict poor outcome in patients, possibly due to provision of migration tracks that facilitate invasion^11,12^. Obtaining these orientation signatures, however, involves imaging on scales that reveal structural organization, and requires image processing in order to reveal quantitative information. Collagen density and stiffness also play a critical role in driving tumor invasion through processes such as increased facilitation of stromal collagen re-organization^13^, modulation of hormone-crosstalk^14^ and activation of mechanically-sensitive signaling pathways^15^. Thus, this apparent significance of collagen and the broader ECM in cancer studies necessitates approaches for quantitative assessment of this specific environment.

Polarimetry is a powerful tool for collagen assessment, since it is sensitive to information about intrinsic structural properties on molecular and fibrillar scales^16,17^, hence presenting an evaluative framework for collagen samples. Polarization investigation has previously been implemented for assessment of both stained^18–20^ and unstained^21–23^ samples, analyzing features such as structural organization, orientation, alignment and birefringence. One approach by Ambekar *et al*.^24^ calculated second-order polarization susceptibility (*χ*^(2)^) matrix elements of collagen using second-harmonic generated intensities. This approach requires the use of a crystallographic model for fitting acquired signals and subsequent estimation of parameters. An alternative polarization framework is Mueller matrix polarimetry which presents a comprehensive polarization methodology since it incorporates the effect of depolarization. Along with other derivable metrics such as diattenuation and retardance, samples can be objectively assessed for evaluation^25^. When combined with imaging^26^, spatially varying polarization information can be extracted for better insight into sample properties^27–29^. As an example, Pierangelo *et al*.^30^ observed that cancerous colon tissues with high cellular density and vascularization depolarized less than non-cancerous tissues, though this was complicated by other factors such as thickness and penetration level in deeper layers. He *et al*.^31^ plotted frequency distributions of Mueller matrix images of cervical tissues, and found larger anisotropy and depolarization in benign tissues compared to abnormal. Following a similar trend, Tata *et al*. also observed lower depolarization in breast cancer tumors grown in mice compared with normal regions^32^, while Dong *et al*.^33^ observed increased linear retardance for *ductal carcinoma in situ* compared with normal tissues.

In the studies highlighted, constituent components in each sample (such as cells, vascularized regions, nuclei, collagen, mucin and lipids) all contribute to the polarization response, and not much is done in separating out individual component polarimetric contributions. As has been motivated earlier, collagen is important for cancer cell development. Hence, it would be beneficial to isolate the polarimetric contributions of collagen, in order to gain an improved understanding of its role during the breast tumor-microenvironment interplay. Second-harmonic generation (SHG) microscopy is a suitable technique for imaging collagenous tissues, but its nonlinearity complicates polarization parameter extraction and interpretation^34–36^. Furthermore, current standard methods employed in diagnostics are mostly tissue consumptive, i.e., the tissue is used up in the process of making diagnosis, tumor phenotyping and prognostic marker determination. Thus, there are ever increasing requirements for more data from sparser tissue. However, biopsy tissue cores are typically extremely slender and apportioning tissue for the various tests is a constant challenge for pathologists. It is desirable to explore different non-consumptive methods to make a binary diagnosis of cancer vs benign.

We have recently developed second-harmonic patterned polarization-analyzed reflection confocal microscopy (SPPARC) microscopy as such a non-consumptive method to obtain the desired discriminatory polarization information in a manner that facilitates intuitive meaning of the measured polarization properties^37^. SPPARC microscopy is able to delineate collagen from other components in a sample, and then extract spatially-dependent linear polarimetric information in three-dimensions from the collagen. In this work, we perform SPPARC imaging on varying pathologies of breast tissues, specifically *benign* (BT), *benign adjacent* (BAT) and *invasive lobular carcinoma* (ILC), in order to tease out differentiating metrics in the relevant collagen ultrastructure and extrafibrillar matrix *plus* cells (EFMC) region. The EFMC, also introduced in the previous work^37^, is the section of the image captured by confocal microscopy but not by SHG microscopy, which implies that this section of tissue is the region with negligible or no collagen. Our study aims to extract intrinsic polarimetric information from tissues in a non-consumptive manner so that scant tissue can be used to obtain therapeutic target and perhaps prognostic information. We highlight parameters that exhibit greatest sensitivity to the different pathologies and present results of variation in these parameters, making comments about possible causes for these differences. To our knowledge, this is the first time that differences between linear Mueller matrix polarization response of collagen in *benign* and *malignant* breast tissues have been explored.

This paper is organized as follows: we briefly present the background of SPPARC microscopy. Next, we show images and results of polarimetric analysis on breast tissues, and comment on the explanation/interpretation of these results. Finally, we discuss current limitations, improvement schemes and future directions in our work.

## Background

The Mueller matrix representation of a sample describes its effect on the polarization state of light, which is captured by the Stokes vector. This relationship is captured by *S*_*o*_ = *M* · *S*_*i*_, where *S*_*o*_ and *S*_*i*_ are the output and input Stokes vectors, respectively, and *M* stands for the Mueller matrix. Retrieving the Mueller matrix involves developing a forward model, and solving the inverse problem using images obtained from different experimental configurations (based on a linear scattering imaging technique such as confocal microscopy).

In order to derive meaning, matrix analysis, decomposition and scalar extraction can then be carried out, yielding optically intuitive parameters such as depolarization, diattenuation and retardance. Depolarization is a measure of how much the tissue randomizes the polarization (geometrical orientation correlations) of the optical field, and this can be a function of thickness, cellular density and molecular structure^30,38^. Diattenuation is a metric that quantifies the preferential absorption of linearly polarized light and finds value in analysis of relatively long or oriented molecules^39,40^, while retardance captures the relative difference in refractive indices along different directions highlighting the anisotropic nature of the tissues^41,42^. Caution should be taken, however, when evaluating retardance, as it can easily be interpreted falsely since there is usually a phase wrapping consideration for tissues thick enough to yield multiple wavelength phase retardation (i.e. if *d*Δ*n* > *λ*, where *d* = thickness, Δ*n* = refractive index difference and *λ* = wavelength of light used). These metrics, along with other derivable quantifiers, can then be used to construct parameter plots, and subsequently lend themselves to analytical or statistical investigation. Two widely used decomposition techniques are Mueller matrix polar decomposition (MMPD)^43^ and differential decomposition (MMDD)^44^.

Second-harmonic generation (SHG) imaging, based on the SHG process, is ideal as a collagen-targeting modality because the *χ*^(2)^ nonlinear susceptibility does not vanish on interaction in the non-centrosymmetric structure of collagen. This is useful as a natural marker for delineating collagenous from non-collagenous regions. Moreover, this provides additional insight when utilized as a complementary technique to other imaging modalities such as two-photon fluorescence^45^, third-harmonic generation microscopy^46^ and quantitative phase imaging^47^.

SPPARC microscopy combines these acquisition/analysis techniques to obtain spatially resolved polarization information in 3D. Briefly, collagen-derived second-harmonic signals in tissue samples are used to pattern a set of polarimetric confocal images, which captures signal from the entire sample. SPPARC analysis provides spatially varying polarization information of collagen in three-dimensions without the need for staining. By simply highlighting regions excluded in the previous process, the non-collagenous extra-fibrillar matrix plus cells (EFMC) can also be quantitatively studied.

## Results and Discussion

**Figure 1:**
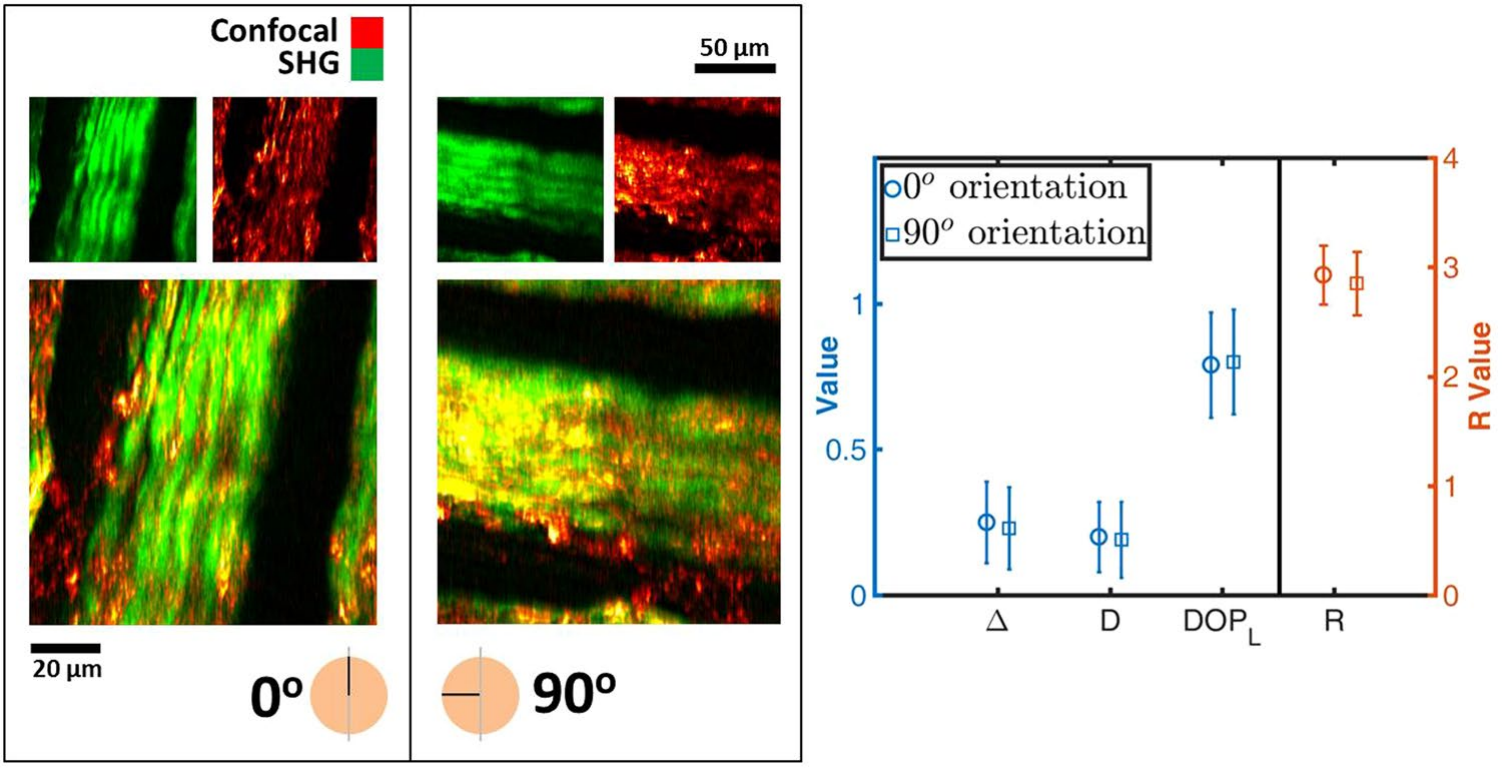
Composite images, with corresponding source SHG (pseudo-colored green) and confocal (pseudo-colored red) images, of orthogonal sample orientations of porcine tendon, used to generate scalar polarimetric parameters. The similarity in error plots between the parameters extracted from both orientations shown on the right, demonstrates the consistent nature of the extracted parameters. Δ – depolarization, D – diattenuation, DOP_L_ – linear degree-of-polarization, R – retardance. The error bars represent the standard deviation of the parameter distribution.

As a first experiment to vet the consistent and intrinsic nature of the scalar polarimetric values extracted by matrix analysis and MMPD, SPPARC microscopy is performed on the same region of porcine tendon placed on the microscope slide holder at orthogonal orientations. It is expected that the intrinsic polarization response parameters would be invariant to rotation, even though the measured Mueller matrices would be different (in fact, the measured matrices should have an orthogonal relationship to each other). Porcine tendon presents a viable sample for verification studies because it is rich in fibrillar collagen^48,49^, thus providing a source of strong optical signals at the fundamental and second harmonic. Figure 1 shows the SHG-confocal image set for a randomly selected region that forms the composite image used to validate the invariance of the polarimetric parameters to rotation. The plot on the right in Fig. 1 shows the mean and standard deviation values of four polarimetric parameters (depolarization Δ, linear degree-of-polarization *DOP*_*L*_, retardance *R* and diattenuation *D*) given the parameter per pixel distribution map derived from SPPARC microscopy. There is close agreement for all the mean values with changes of 8% (0.25,0.23), 1% (0.79,0.80), 3% (2.93,2.85) and 5% (0.20,0.19) for Δ, *DOP*_*L*_, *R* and *D* respectively.

A Pearson product-moment correlation is computed to quantify the relationship between data obtained in the different configurations. We calculate positive correlation between configurations for all variables (*p* ≈ 0.70, 0.68, 0.91, 0.89 for Δ, *D*, DOP_L_, *R*, respectively). The approximately equal standard deviation values and positive correlation demonstrate the consistency in parameter variation across the field-of-view considered. It is noted that the Δ and *D* correlation values are not as high as those for DOP_L_ and *R*. This could be attributed to the fact that we manually take out and rotate the sample during experiments, thereby introducing some variability in experimental conditions and offset in image registration. However, our subsequent experiments in this work only use a single configuration, obviating the need to repeatedly take the sample out.

**Figure 2:**
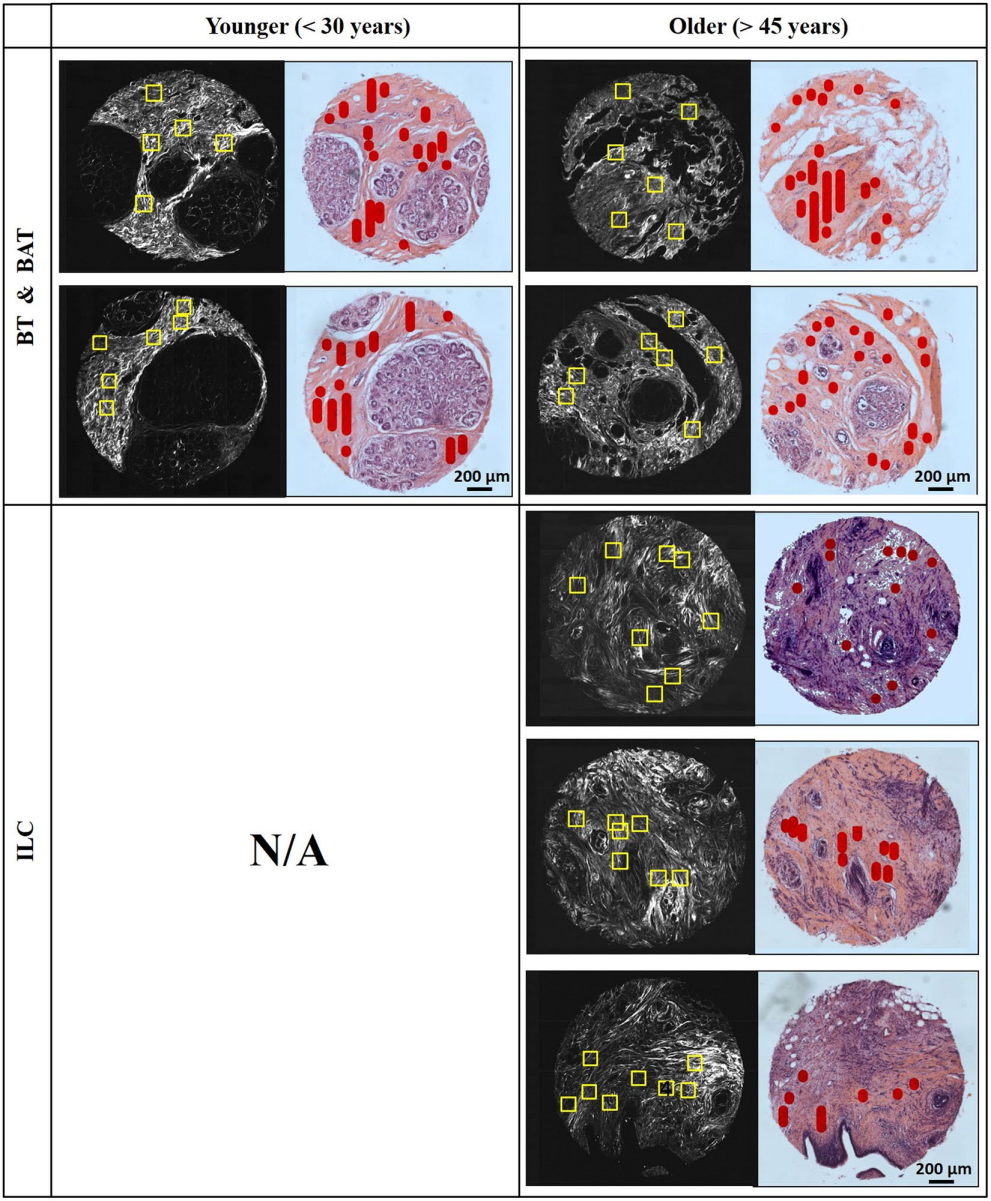
SHG (black-and-white) and H&E stained (color) images of consecutively cut sections of breast TMA cores from *benign tissues* (BT), *benign adjacent tissues* (BAT) and *invasive lobular carcinoma* (ILC). Regions of interest marked in red in the H&E images guide the selection of regions (47 in all) which undergo SPPARC analysis (marked by yellow boxes in the SHG images).

**Figure 3:**
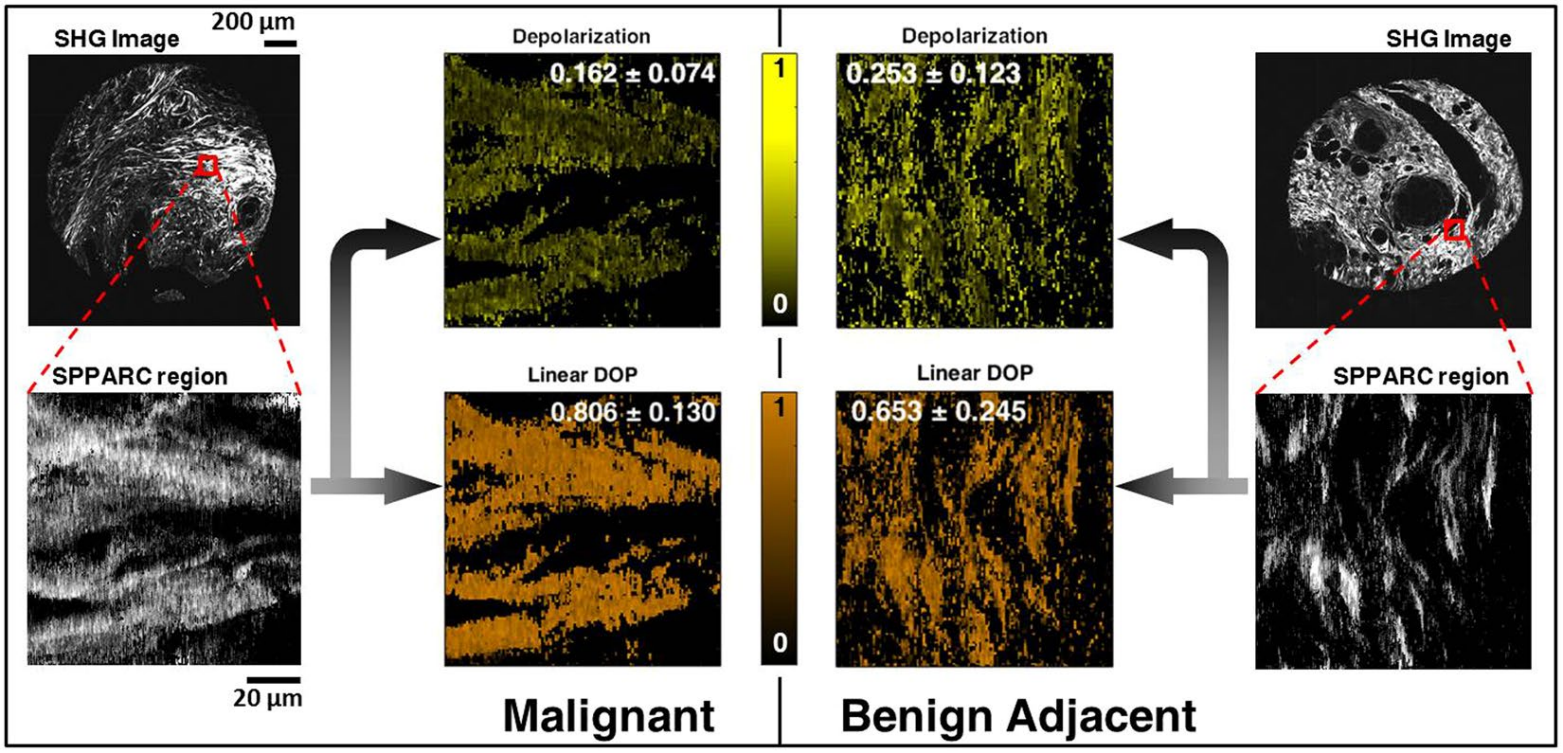
Images of one selected region each from two TMA sample cores: one from an ILC core top left) and another from a BAT core (top right). SPPARC images for selected regions with stromal collagen within tumor for ILC and stromal collagen in benign tissue adjacent to tumor for BAT are shown. The corresponding depolarization and linear DOP parameter spatial maps are also shown (along with inset text showing mean and standard deviation over all pixels in the image) revealing a lower depolarization and higher linear degree-of-polarization for stromal collagen within tumor.

We next proceed to perform imaging and analysis on breast tissues. A breast tissue micro-array (TMA) with regions designated above as BT and BAT as control, along with ILC is assessed using SPPARC microscopy. Each tissue core has a diameter of ~1.5 mm and is ~25 *μ*m thick. More details about the breast TMA are given in the Methods section. Figure 2 shows SHG images and the corresponding brightfield images of consecutively cut H&E stained cores for BT, BAT and ILC selected from the TMA. The age classification for the cores is also shown, with ILC and BAT obtained from women older than 45 years, while BT samples were obtained from women younger than 30 years. Stromal regions of interest on the tissue cores considered are marked in red on the H&E brightfield images, and used as a reference to perform SPPARC imaging on relevant sections (highlighted with yellow boxes in the SHG images). We compare the perilobular stroma of BT and the available stroma adjacent to BAT and surrounding/within ILC. We also avoid regions which display observable shrinkage and other artifacts due to tissue processing. Figure 3 shows SHG images of a BAT core and an ILC core, along with ~100 *μ*m regions selected for SPPARC microscopy. As described in detail in our previous work^37^, parameter spatial map distributions for these regions are generated, and the results for two of these parameters, depolarization and linear degree-of-polarization, are shown. In these representative examples, it is observed that the depolarization effect (mean ± standard-deviation over the image pixels) of the stroma within malignant tissue (0.162 ± 0.074) is less than that of the BAT (0.253 ± 0.123). Thus, the collagen contribution in malignant tissues shows a similar trend of the lower mean depolarization reported in previous studies of tumors^30–33^. In contrast, the effective linear degree-of-polarization of the output light after interaction with the collagen in samples shows the opposite trend (0.806 ± 0.130 and 0.653 ± 0.245, respectively), agreeing with work that highlights DOP_L_^50,51^.

**Figure 4:**
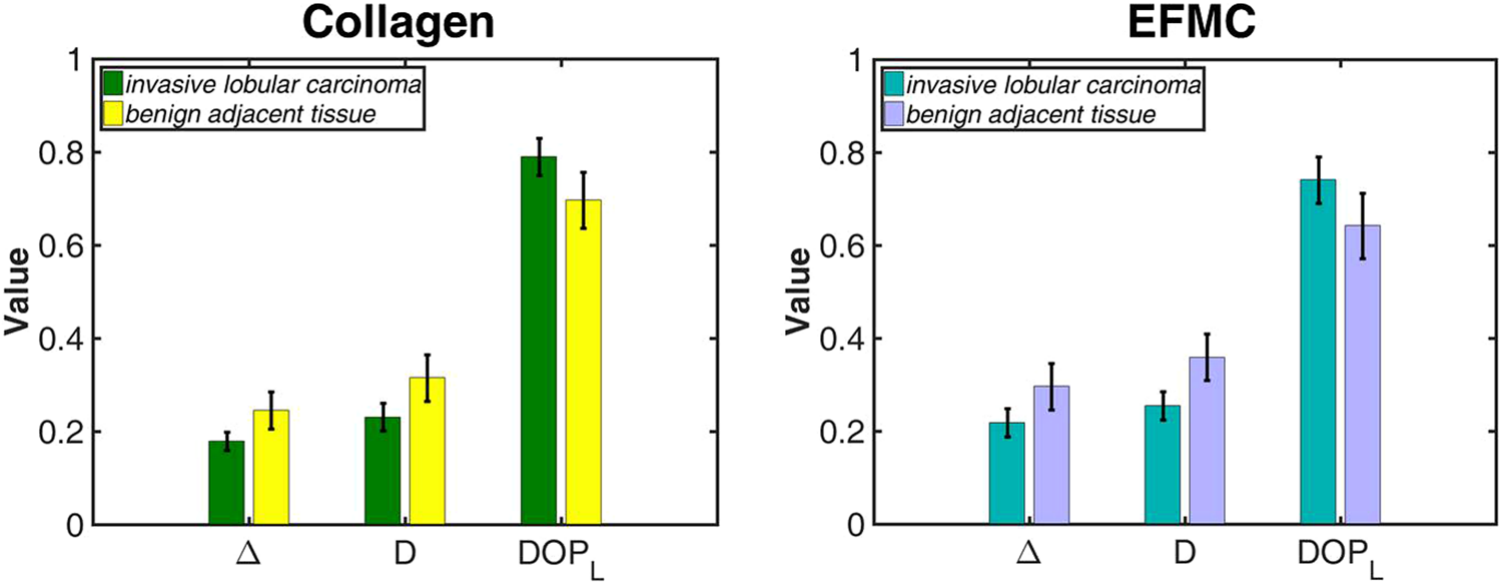
Plots of parameter mean with standard deviation error bars from all regions imaged, comparing stromal collagen within tumor (ILC) with stromal collagen in benign tissue adjacent to tumor (BAT) for both collagen and the EFMC. The two plots exhibit similar trend of lower depolarization, lower diattenuation and higher linear degree-of-polarization for stromal collagen within malignant tissue.

For increased statistical strength, SPPARC microscopy is performed on 47 regions comprising 23 ILC and 24 benign regions (11 BT and 13 BAT sections). We find that the greatest parameter differences from collagen in ILC come from those in BAT sections. This suggests that the collagen optical properties change most in the vicinity of tumors. We highlight in Fig. 4, the error bar comparisons of different parameters between collagen and the EFMC in BAT and ILC (the full plots showing comparison between BT, BAT and ILC are shown in the Supplementary Information). An independent-samples *t*-test is carried out to compare selected polarization metrics between BAT and ILC for both collagen and the EFMC. It is observed that for collagen, there are significant differences in the comparison of parameters Δ (*p* ≈ 0.001), *D* (*p* ≈ 0.047) and DOP_L_ (*p* ≈ 0.001) given a significance level of 0.05. Significant differences are also observed for the EFMC comparison yielding *p* values of 0.002, 0.028 and 0.002 for Δ, *D* and DOP_L_ respectively. As previously observed in the analysis of a single pair of regions, stromal collagen within malignant tissues show average lower Δ values than in BAT. We hypothesize that this may be due to the fact that malignancy induces less optical scattering, and hence less polarization decoherence, as a result of stiffening of the surrounding stromal collagen. Stromal stiffening is an effect of increased matrix deposition leading to cross-linking in the tumor micro-environment^15,52,53^. A similar trend of reduced *D* for stromal collagen within ILC implies a reduction of preferential absorption of incident polarized light. We propose that this may be due to the loss in general optical anisotropy in stromal collagen^54^, influenced by the haphazard growth and invasive behavior of the tumor. DOPL shows the reverse trend, with stromal collagen in ILC preserving linear degree-of-polarization more than collagen in BAT. The overall stromal collagen relationship is also noted for the EFMC, although there appears to be slightly higher spread in the parameter variation as captured by the standard deviation. In the retardance (R) data obtained for both collagen and the EFMC, there was significant overlap between the error bars. Thus as a differentiating metric, its relevance was minimized by this overlap, and hence it was not included.

It is important to note that the stromal collagen regions around malignant tissues assessed are themselves not classified as malignant. However, we do note differences in the polarization response depending on whether the collagen is in the vicinity of the tumors, adjacent to tumors or in benign tissues. When the malignancy arises from stromal collagen and other mesenchymal tissues, it is referred to as sarcoma^55,56^, which is not the case we believe to have here. Another important point is that the stromal collagenous regions in BT were obtained from women younger than 30 years, while those for BAT and ILC were obtained for women older than 45 years. This creates a potential multivariate effect in the data for BT, and hence, may require further studies to see its effect. The comparison plot for BT is included in the Supplementary Information.

## Summary

In conclusion, we experimentally demonstrated the consistency of SPPARC analysis, and compared metrics between perilobular stroma in benign mammary tissue, and the available stroma adjacent to and surrounding malignant *invasive lobular carcinoma*. To our knowledge, this is the first time that such comparison of the linear Mueller matrix polarization response targeting stromal collagen in *benign* and around *malignant* breast tissues has been done. The most noticeable differences in metrics occur between BAT and stromal tissue around malignant tissues, where depolarization, diattenuation and linear degree-of-polarization appear to be sensitive to tissue pathology. A key observation is that stroma surrounding malignant tissues show lower depolarization, lower diattenuation and higher linear degree-of-polarization than stroma adjacent to malignant tissues. These results pave the way for an expanded study involving more varied pathologies, and multivariate analysis that incorporates age, disease stage and more pathology diagnoses.

As a label-free and minimally consumptive method, SPPARC can be used as an imaging tool for nondestructive, quantitative assessment of collagen in tissue samples. In addition to providing deeper understanding about the biological processes underlying disease progression, it can help as a complementary technique in a clinical setting for comprehensive analysis. By separating out the effects of collagen changes, it is possible to more accurately specify root-cause effects that can help with prognosis and therapy. A current limitation is the data acquisition time for SPPARC microscopy. The process is currently being optimized for faster acquisition. In addition, experiments suggest that the parameter sensitivity increases with sample thickness, and hence use of thicker samples will enhance the differences in polarimetric effect, and aid in more accurate distinction of pathologies. Furthermore, a potentially interesting study would be a comparison of the polarization response between the briefly highlighted sarcoma and benign stromal collagen.

## Methods

### Porcine tendon sample

Porcine tendon, extracted from pig feet purchased from the local market, was embedded in optimal cutting temperature (OCT) compound at −25 °C, cut into thin sections using a cryostat and soaked in 1× phosphate-buffered saline to remove excess OCT. The sections were next placed on microscope slides, and gently held in place with coverslips using aqueous mounting media. The experiment with porcine tendon is exempt from the Illinois Institutional Animal Care and Use Committee and was carried out in accordance with relevant guidelines and regulations.

### Human breast tissue microarray (TMA)

The human breast TMA used in this study (labeled T088b) was purchased from US Biomax, Inc. The TMA specification obtained from the manufacturer includes age, grade, type and pathology diagnosis. Consecutive 25 *μ*m sections of each tissue core were cut, and one set stained with hematoxylin and eosin in order to obtain corresponding brightfield images to the SHG images of the unstained second set. This is done so as to easily highlight regions of interest in the H&E stained images that will be assessed with SPPARC on unstained sample. The experiments on the TMA public use data are exempt from review by the University of Illinois Institutional Review Board. All experiments were carried out in accordance with relevant guidelines and regulations.

### Experimental setup

Brightfield images of the cores are acquired using a Zeiss observer Z1 inverted microscope, as described in the Methods section of ref.^57^. Light from a halogen lamp source illuminates the sample, and the scattered light, collected by a 10× Plan-Neofluar objective, is imaged to an Axiocam 503 color CCD camera. SHG imaging of the core samples is performed with a Zeiss NLO 710 microscope attached to an 80 MHz laser source as described previously^58,59^. The SPPARC microscopy setup for collagen Mueller matrix polarimetry has also previously been described in detail^37^. Briefly, laser light at a wavelength of 780 nm from a 100 fs pulsed, 80 MHz repetition rate source is scanned using galvo mirrors and guided towards a strain-free 40× 0.65 NA objective lens which focuses the light onto the sample plane. Epi-directed SHG signals at 390 nm and linearly scattered reflection confocal signals at 790 nm are collected by the same objective, partly reflected by a beam splitter and relayed to a photomultiplier tube (PMT) detector. A 50 *μ*m confocal pinhole-PMT arrangement is used to collect the linearly scattered signals, while the SHG signals are collected by a combination of SHG filter (18 nm bandwidth centered at 390 nm) and PMT. A polarization state generator (PSG) and polarization state analyzer (PSA), each comprising reverse arrangements of linear polarizer, half-wave plate and quarter-wave plate, set up the different optical polarimetric configurations for generation and analysis of polarization states. For a given region, a complete set of oversampled polarization information (thirty-six 256 × 256-pixel images) is acquired in ~1 hour. Each acquired image has a pixel size of ~400 nm.

### Mueller matrix analysis

Each polarimetric confocal image is segmented using an SHG mask. This SHG mask, generated by binarizing the source SHG image using a previously set threshold, is used to delineate the acquired confocal images. In this way the collagen-rich regions above an SHG signal threshold, and the non-collagenous regions (referred to as extracellular matrix *plus* cells, EFMC) below this threshold can be decoupled. Spatial polarization information using matrix analysis and MMPD across an image is obtained per 2 × 2 binned pixels, in order to mitigate the effect of pixel saturation that may occur randomly during signal acquisition and for faster analysis.

## Electronic supplementary material

Supplementary Information
