# Supplementary material for: Second-harmonic patterned polarization-analyzed reflection confocal microscopy of stromal collagen in benign and malignant breast tissues
Source: Sci Rep. 2018 Nov 2;8:16243. doi: 10.1038/s41598-018-34693-0 (PMC6214917; doi:10.1038/s41598-018-34693-0)
Supplement: Supplementary file 1 — Supplementary Information [file 41598_2018_34693_MOESM1_ESM.docx]

Supplementary Information

**Second-harmonic patterned polarization-analyzed reflection confocal microscopy of stromal collagen in benign and malignant breast tissues**

# Chukwuemeka Okoro1, Varun Kelkar1, Mayandi Sivaguru2, Rajyasree Emmadi3, and Kimani C. Toussaint, Jr.4,5,*

*1University of Illinois at Urbana–Champaign, PROBE Lab, Department of Electrical and Computer Engineering, Urbana, Illinois, 61801, USA*

*2University of Illinois at Urbana–Champaign, Carl R. Woese Institute for Genomic Biology, Urbana, Illinois, 61801, USA*

*3University of Illinois at Chicago, Department of Pathology, College of Medicine, Chicago, Illinois, 60612, USA*

*4University of Illinois at Urbana–Champaign, PROBE Lab, Departments of Mechanical Science and Engineering and Bioengineering, Urbana, Illinois, 61801, USA*

*5University of Illinois at Urbana–Champaign, Affiliate in the Departments of Electrical and Computer Engineering, and the Beckman Institute for Advanced Science and Technology, Urbana, Illinois, 61801, USA*

***[*Corresponding author: Kimani C. Toussaint, Jr. ktoussai@illinois.edu*](mailto:ktoussai@illinois.edu)

# 1. Extrafibrillar Matrix *plus* Cells (EFMC) regions


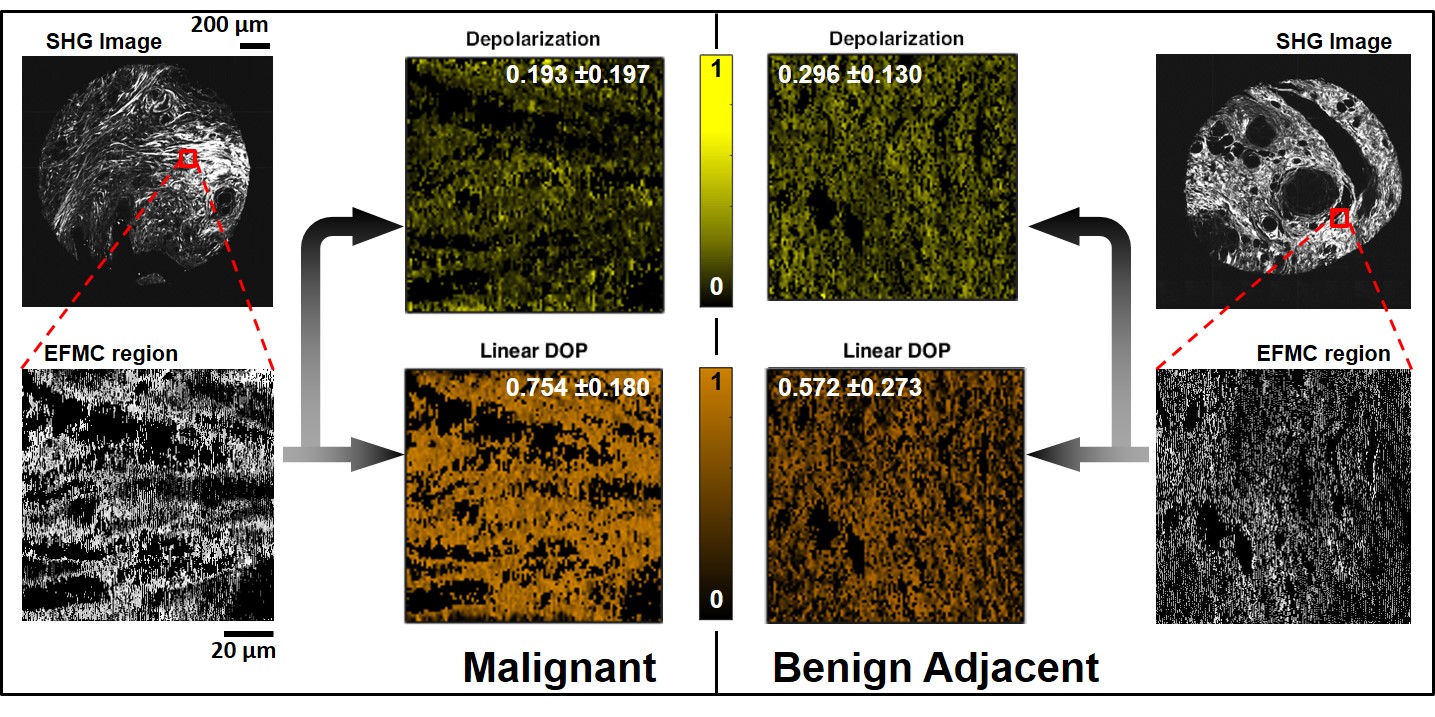


**Supplementary Figure 1**: Images of one selected region each from two TMA sample cores: one from an ILC core top left) and another from a BAT core (top right). EFMC images for selected regions that exclude stromal collagen in tumor for ILC and stromal collagen in benign tissue adjacent to tumor for BAT are shown. The corresponding depolarization and linear DOP parameter spatial maps are also shown (along with inset text showing mean and standard deviation over all pixels in the image), revealing a lower depolarization and higher linear degree-of-polarization for stromal collagen within tumor.

Figure 1 shows SHG images of a BAT core and an ILC core, along with ~ 100 µm regions selected for SPPARC microscopy. The parameter spatial map distributions for these regions are generated for the EFMC regions, and the results for two of these parameters, depolarization and linear degree-of-polarization, are shown. As in the collagenous regions results shown in the main text, it is observed that the depolarization effect of the EFMC within malignant tissue (0.193±0.197) is less than that of the BAT (0.296±0.130). The reverse trend is shown for the linear degree-of-polarization (0.754±0.180 and 0.572±0.273, respectively).

# 2. Parameter Plots for Three Pathologies


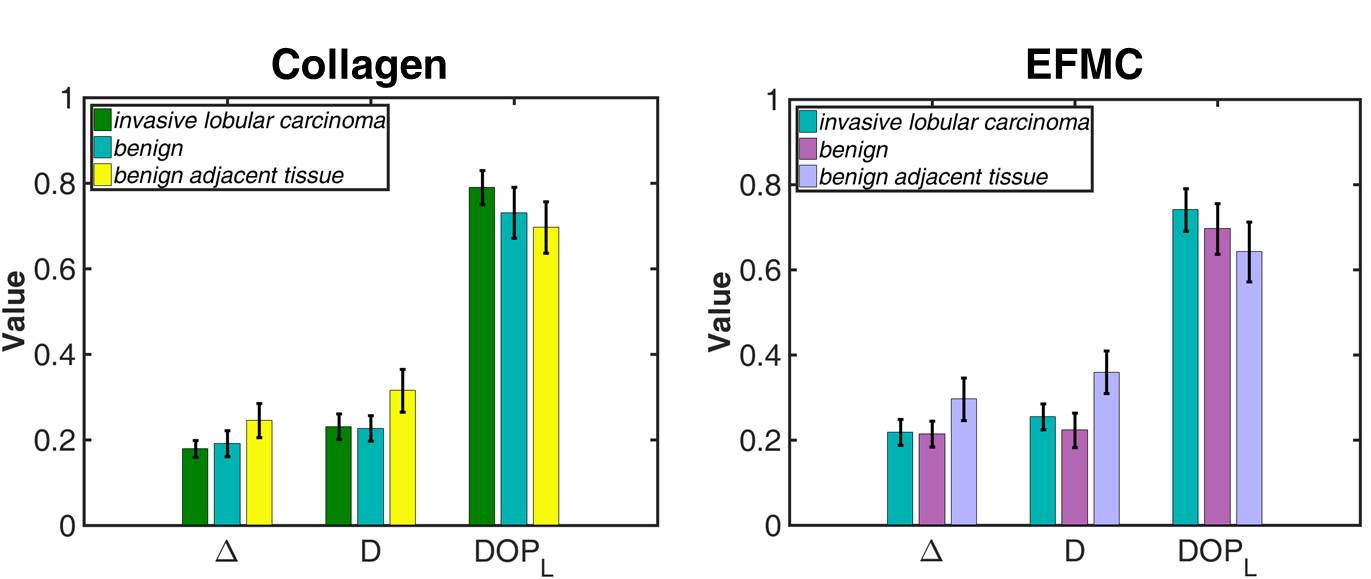


**Supplementary Figure 2**: Plots of parameter mean with standard deviation error bars from all regions imaged, comparing stromal collagen within tumor (ILC), stromal collagen in benign tissue adjacent to tumor (BAT) and stromal collagen in benign tissue (BT) for both collagen and the EFMC.

Figure 2 shows the full comparison of depolarization (Δ), diattenuation (D) and linear degree-of-polarization (DOP_L_) values for perilobular stroma in benign mammary tissue (BT), and the available stroma adjacent to (BAT) and surrounding/within the malignant invasive lobular carcinoma (ILC). We observe minimal difference between stromal collagen in BT and ILC, as opposed to more observable differences from BAT.
